# Development of Whole Genome SNP-CAPS Markers and Preliminary QTL Mapping of Fruit Pedicel Traits in Watermelon

**DOI:** 10.3389/fpls.2022.879919

**Published:** 2022-05-09

**Authors:** Sikandar Amanullah, Benjamin Agyei Osae, Tiantian Yang, Shenglong Li, Farhat Abbas, Shi Liu, Shusen Liu, Zhengfeng Song, Xuezheng Wang, Peng Gao, Feishi Luan

**Affiliations:** ^1^College of Horticulture and Landscape Architecture, Northeast Agricultural University, Harbin, China; ^2^Key Laboratory of Biology and Genetic Improvement of Horticulture Crops (Northeast Region), Ministry of Agriculture and Rural Affairs, Harbin, China; ^3^College of Horticulture, South China Agricultural University, Guangzhou, China; ^4^Shouguang Sanmu Seed & Seedling Co., Ltd., Shouguang, China

**Keywords:** watermelon (*Citrullus lanatus* L.), fruit quality, fruit pedicels, genetic markers, linkage map, QTL

## Abstract

Fruit pedicel (FP) is an important determinant of premium fruit quality that directly affects commercial market value. However, in-depth molecular and genetic basis of pedicel-related traits has not been identified in watermelon. Herein, a quantitative trait locus (QTL) mapping strategy was used to identify the potential genetic regions controlling FP traits based on newly derived whole-genome single nucleotide polymorphism based cleaved amplified polymorphism sequence (SNP-CAPS) markers. Next-generation sequencing based whole-genome re-sequencing of two watermelon parent lines revealed 98.30 and 98.40% of average coverage, 4,989,869 SNP variants, and 182,949 CAPS loci pairs across the reference genome, respectively. A total of 221 sets of codominant markers exhibited 46.42% polymorphism rate and were effectively genotyped within 100-F_2:3_ derived mapping population. The developed linkage map covered a total of 2,630.49 cM genetic length with averaged 11.90 cM, and depicted a valid marker-trait association. In total, 6 QTLs (*qFPL4.1, qFPW4.1, qFPD2.1, qFPD2.2, qFPD8.1, qFPD10.1*) were mapped with five major effects and one minor effect between the whole genome adjacent markers positioned over distinct chromosomes (02, 04, 08, 10), based on the ICIM-ADD mapping approach. These significant QTLs were similarly mapped in delimited flanking regions of 675.10, 751.38, 859.24, 948.39, and 947.51 kb, which collectively explained 8.64–13.60% PVE, respectively. A highly significant and positive correlation was found among the observed variables. To our knowledge, we first time reported the mapped QTLs/genes affecting FP traits of watermelon, and our illustrated outcomes will deliver the potential insights for fine genetic mapping as well as functional gene analysis through MAS-based breeding approaches.

## Introduction

Watermelon (*Citrullus lanatus* L.) is a diploid (2n = 22) flowering plant, with trailing vine-like growth habit that needs a long growing season (Zhang et al., 2021). It is a commercially cultivated member of the Cucurbitaceae family, sold in fresh-cut form, and primarily consumed as a juicy summer fruit around the world. China alone produces 60.7 mega tons (Mt) of watermelon which is two-thirds (60%) of the worldwide production, followed by Turkey, India, and Iran, respectively (Grumet et al., 2021). Watermelons are thought to be native to north-eastern Africa. The taste of wild type watermelon was bitter and the flesh was yellowish-white during its earlier domestication. Then, considerable multiple breeding efforts made the effective possibilities for the genetic improvement of watermelon. It also represents a remarkable diversity in fruit related phenotypes (Pan et al., 2020), by specifying the culinary, aesthetic, as well as medicinal values.

Each plant organ has its own importance and is truly connected with each other in a direct or indirect way. The pedicel “also known as stalk” is a first emerging short-stem organ that connect the flower, ovary, and fruit to their primary inflorescence axis attached with the main plant body (Setiamihardja and Knavei, 1990). The internal structure of pedicel contains vascular bundles (xylem and phloem) and pith cells (spongy ground tissues) (Douglas and Riggs, 2005), that mainly act as a bridge for transportation of photosynthate, and intake of water, minerals, and nutritions. Further, the structural properties significantly affect the fruit pedicel (FP) traits (length, weight, and diameter) which exhibit a direct contribution for developing the good-quality fruits. The non-uniform FP affects the fruit's outer appearance and similarly provides bitterness due to the irregular growth of fleshy tissue structure (Che and Zhang, 2019).

In general, FPs are known to be used as a traditional Chinese medicine, e.g., muskmelon base termed “Pedicellus Melo.” is consumed to treat edema and coughs (Gao et al., 2012). Two main medicinal components of FP are cucurbitacin D and cucurbitacin B, which are the primary bioactive derivatives from muskmelon pedicel base (Edery et al., 1961; Hu et al., 1982). These derivatives inhibit the growth of breast cancer (Wakimoto et al., 2008), laryngeal squamous cell carcinoma cells (Liu et al., 2008), myeloid leukemia (Haritunians et al., 2008), and also exhibit *in vitro* hepatoprotective activity (Bartalis and Halaweish, 2011). The lengthy FPs are more suitable for medicinal uses because they provide a greater yield of relevant medicinal ingredients (Gao et al., 2012). However, FP characteristics vary in each fruit crop, e.g., from short to long (1–10 cm) in cucumber (Song et al., 2016), from 2.5 to 20 cm in muskmelon (Cui et al., 2022), soft and hard, thin and thick, light and heavy pedicel in pepper (Solomon et al., 2021).

In addition, immature FPs are tender and easily broken which causes pre-harvest fruit dropping, and ultimately this damage turns into post-harvest loss in yield, shelf life, and transportability (Fanourakis and Tzifaki, 1992). The mature FPs play a potential role in developing the premium fruit quality and primarily determine the average yield (Zhao et al., 2015). In China, fresh and premium-grade marketable cucumbers should account for <14% of the total fruit length (1/7 part) (Zhou et al., 2005). Further, mature lengthy pedicel with more diameter can grasp the large and heavy weighted fruits (Solomon et al., 2021) because endogenous lignification gradually modifies the cell tissue structure which led to stem hardness (Yang et al., 2022). In cucumbers, the key to efficient mechanical harvesting depends upon the differentiation between mature FPS, leaves, and main stems; however, lengthy pedicel provides more feasibility for this kind of procedure by proper gripping, and detachment of fruit from the pedicel (Song et al., 2016). Thus, fruit pedicel morphology directly indicates the quality of the fresh market and efficient harvesting.

Fruit direction/orientation also depends upon the FP and its related traits, and also exhibit a great connection with the fruit size and weight (Setiamihardja and Knavei, 1990). The straight and curved type growth of FP directs the proper fruit placement in erect or pendent form, respectively (Paran and Van Der Knaap, 2007; Solomon et al., 2021). This mechanism was known to be synchronized by a controlled and sequential cell proliferation, division, and elongation (Douglas et al., 2002; Bundy et al., 2012). A study of pedicel development in *Arabidopsis* also specified the preliminary key signs of mechanisms by which the proximal constriction of pedicel at abaxial and lateral sides triggers the downward bending of the distal pedicel (Douglas and Riggs, 2005). In cucumber, the additive genetic effect of fruit neck length (FNL) accounted for 97.9% phenotypic variation, signifying more genetic variability effects rather than environmental variability (Gu et al., 1994). The genetic inheritance of FNL in cucumbers is due to a mixed and major polygenic architecture (Ma et al., 2010; Chen, 2011). However, the genetic basis studies of FP traits have not been fully confirmed yet.

With the advent of new omics technologies, NGS-based whole-genome re-sequencing has emerged as a multiplexed, quick, and reproducible method after publishing the first-ever sequenced genome of watermelon (Guo et al., 2013). This molecular approach estimated the watermelon genome size (~425 Mb) and further sequencing also annotated a total of 23,440 predicted different types of genes encoding protein and mRNA in the whole genome assembly (Guo et al., 2019). Genome-wide NGS similarly proved to be an efficient technique for quickly understanding the in-depth genetics of complex genomic assets of watermelon, and the possibilities of generating high density markers were also increased (Liu et al., 2016). This technique deeply identifies several potential genetic variants (Amanullah et al., 2018), as well as novel markers based genetic loci mapping (Amanullah et al., 2020).

Quantitative trait locus mapping is a potent conventional strategy which is utilized for the detection of candidate genomic regions regulating various traits, and closely localized markers can be applicable in marker-assisted selection (MAS) based molecular breeding (Amanullah et al., 2021). In the last decade, whole genome SNP-derived CAPS markers have emerged as potential genetic markers for uncovering the genetic regions affecting complex types of qualitative and quantitative traits in differentially derived populations of unexplored botanical groups. For example, powdery mildew resistance (Li et al., 2017), stripped fruit skin (Liu et al., 2019), ovary-fruit associated morphology (Amanullah et al., 2018, 2020, 2021), plant height (Zhang et al., 2021), pale green and dark red color flesh (Wang et al., 2019; Pei et al., 2021), egusi seed traits (Osae et al., 2021), and fruit peel hardness (Yang et al., 2021).

Not all the genetic components have been dissected in all of the unexplored cultivars/groups. To our knowledge, genetic mapping of FP traits of watermelon is still unknown and needs to be understood in detail. Therefore, we first time reported this study aimed at the development of SNP-CAPS markers and preliminary QTL mapping of the FP traits of watermelon by utilizing a newly derived biparental F_2:3_ population. We believe that the current study results would be useful in providing potential genetic insights for further MAS-based molecular and genetic breeding methods.

## Materials and Methods

### Experiment Materials and Growing Conditions

**Figure 1:**
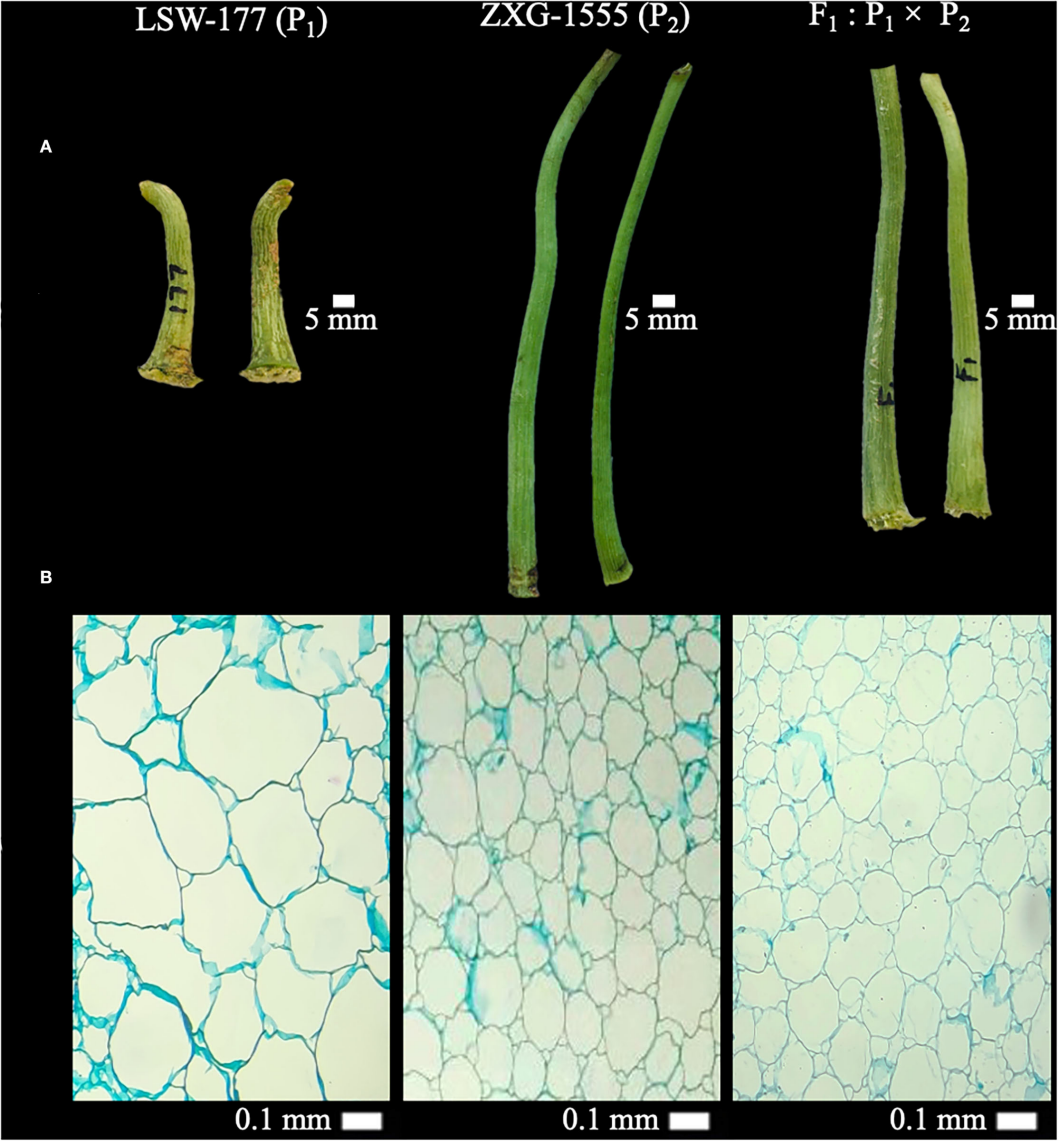
Primary phenotypes and cytological observations of fruit pedicels (FP) of two watermelon parent lines and F_1_ progeny. **(A)** Phenotypic observations at 5 mm scale. **(B)** Cytological observations at 40× magnification (0.1 mm scale) of light microscope.

Two watermelon parent lines “LSW-177 (female as P_1_, short-sized FP with less weight, thick diameter, and big cell structure in pith) and ZXG-1555 (male as P_2_, lengthy FP with more weight, thin diameter, and small cell structure in pith)” were chosen as experimental materials on the basis of their primary phenotypes. The cytological and morphological differences of FP traits among both parent lines and their resultant genetic populations are shown respectively (Figure 1, Supplementary Figure 1).

A field experiment was conducted in the big plastic greenhouse (600 × 300 m^2^) at the XiangYang Experimental Base of Northeast Agricultural University (NEAU), Harbin (44°04′ N, E125°42′), China. In 2020, whole genome re-sequencing-based bio-informatic analysis were performed between two contrasted parents (P_1_ and P_2_) and novel SNP-derived CAPS markers were developed. These parents were also crossed to produce the F_1_ progeny, and this F_1_ was further self-pollinated to develop the respective mapping populations of 100-F_2_ derived F_3_ families.

In 2021 year, the seedlings of all genotypes were raised and 100-F_3_ families (5 plants per family) were cultivated in a complete randomized design (CRD), along with P_1_ (15 plants), P_2_ (15 plants), and F_1_ progeny (15 plants), respectively. The plants were cultivated at 65 cm apart with rows that were 45 cm apart, and standard cultivation practices were adopted to promote the healthy growth of plants and decrease the chances of major disease attacks, etc.

### Phenotyping of Fruit Pedicel Traits

FPs of F_3_ families were collected during the harvesting of a commercial fruit crop of watermelon at days after pollination (DAP) and maturity signs. The weight of FP (FPW) was freshly calculated using a digital weighing scale, and values were recorded in grams (g). Then, FPs were manually straightened and pedicel length (FPL) was measured. FP diameter (FPD) was checked with a phenotypic precision of thickness from the upper, middle, and lower positions. FPL and FPD were measured with a digital vernier caliper and data values were recorded in mm.

### Histology of Fruit Pedicels

The freshly harvested FPs were sampled, dissected in longitudinal form, and 1 cm paraffin-embedded sections were prepared. All samples were then fixed in an equal fixative solution of 3.5% formaldehyde, 5% glacial acetic acid, and 50% ethanol for complete embedding. A marked series of ethanol (70, 85, 95, and 100%) was used for the ultra-dehydration of fixed samples followed by the xylene and ethanol ratio. A total of 10-μm microtomes were prepared, stained by using Toluidine Blue, and examined at 40 × magnification (0.1 mm scale) of optical microscope as mentioned (Yang et al., 2021). The total cell numbers of each pedicel sample were photographed and visually checked by using the digital scale bar of image processing software “ImageJ” (https://imagej.nih.gov/ij/).

### GDNA Isolation and Whole-Genome Re-sequencing

The leaf material (0.2 g) was taken from two-week-old seedlings of all cultivated genotypes, snap-frozen with cryogenic liquid nitrogen, and preserved at an ultra-high-temperature of −80°C. A good quality gDNA was extracted using the cetyl trimethyl ammonium bromide (CTAB) protocol with slight modifications (Allen et al., 2006). The quantified gDNA was fragmented with Bioruptor (Thermo Fisher Scientific, USA) and 200–300 bp fragments were pooled-up. A sequencing library of each parent line gDNA was then prepared as follows: (1) the gDNA fragments were subjected to end-repairing and A-tailing; (2) the resulting fragments were ligated with bubble adapters that contained a sequential barcode, and then amplified with polymerase chain reaction (PCR). Next-generation sequencing (NGS, High-throughput Illumina Sequencing^TM^ 2500) based whole genome re-sequencing (>25× genome depth coverage) was performed at Beijing Genomics Institutes (BGI) company, Shenzhen, PR China.

### Mapping of Re-sequencing Reads

The total re-sequencing data of each watermelon line were quality-checked using the pre-processing FASTX-Toolkit v0.0.13. We first filtered the raw/low-quality adapter sequences, then went through a series of data processing for quality trimming, and obtained the valid data of clean read bases by using the filter parameters of SOAPnuke software (developed by BGI) as follows: (1) the reads that match > 25% of adapter sequences were deleted for filtered adapter, (2) the reads that match more than > 50% of bases having a quality value of <20 were removed, and (3) then reads with > 3% N were removed. The resultant clean end bases of paired-end sequencing of both parent lines were aligned to the de novo assembled reference genome (v2) of watermelon (97103) using the Burrows-Wheeler Aligner (BWA)-MEM algorithm (https://sourceforge.net/projects/bio-bwa/files/) (Li and Durbin, 2009). The Binary Alignment/Map (BAM) files were similarly used for fixing the filtered alignment of re-sequenced bases, and marked duplicate bases were discarded using the Picard tools (v1.106), and the finally sorted BAM sequences were used for subsequential analysis.

### SNP Variants Calling and Annotation

Whole-genome SNP variants were called by using the Soaps v.1.03 (Tian et al., 2009), the Sequence Alignment/Map format of SAMtools software package v1.14 (http://www.htslib.org/download/) (Li et al., 2009), and Genome Analysis Toolkit (GATK) v4.2.0.0 (https://www.broadinstitute.org/gatk/) (McKenna et al., 2010). This program allows the local alignment and calibration to generate the SNP variants per sample in VarScan2 file format. The output file format consisted of indexed information related to the positions of variants along with coverage of quality mapping and in-depth read statistics (Ruffalo et al., 2011).

All the filtered SNP variant call format (VCF) were annotated based on their physical positions, and categorized according to their genetic variation using SnpEff v4.3 (Cingolani et al., 2012). The binary (.bin) database of reference annotated watermelon genome files (.gff3) and genomic sequences were downloaded and aligned for annotation of genetic variant effects by their regions (intergenic and exonic) and impact (high, low, moderate, and modifier), and similarly classified by their functional classes (missense, non-sense, and silent), respectively. The web-based generated HTML and VCF files were used for identifying the genetic locations of introns, exons, start-stop codons, upstream-downstream regions, splice regions, and 5' to 3' end UTR regions.

### Identification of SNP-CAPS Loci

Whole-genome SNP-CAPS loci pairs were identified using the following steps as mentioned in our earlier study (Amanullah et al., 2020). Firstly, putative SNPs sequences were detected from the mapped re-sequencing reads by setting the default protocol of BWA and SAM tools, respectively. Then, flanking sequences with >20 quality mapping scores and 500 base pairs (bp) were pair-wise filtered at each SNP locus. The frequency of finally retrieved SNP-CAPS loci pairs were analyzed by the self-compiled perl script and SNP2CAPS tool, with an input parameter setting and required cutting sites of different endonucleases (Thiel et al., 2004), and the further missing CAPS loci were also retrieved through the same procedure by using SnapGene viewer (v6.0). Finally, a total of 40–45 suitable CAPS loci pairs were used for primer designing and oligo synthesizing.

### Marker Development and PCR Validation

The physical positions of whole-genome SNP-CAPS loci pairs were cross-checked and PCR primers (forward and reverse sequences) were developed using the latest primer designing tool “Primer Premier (v6.20)” as mentioned (Amanullah et al., 2020). The default preferred parameters were as followed; optimal length of 18–22 bp, primary melting temperature (T_m_) of 50–60°C and 3°C gradients in secondary annealing (T_a_) temperature, GC contents of 40–60%, and amplification length of >500 bp. The exported primers were then marked with a unique ID and abbreviated as “chromosome number, respective name of cutting site endonuclease, and a number of markers on each chromosome”, respectively.

The oligo-synthesized primers were preliminary screened using the optimized polymerase chain reaction (PCR) in a 20 μL mixture as mentioned (Amanullah et al., 2021). Then, the exact polymorphism was cleaved by performing the enzyme digestion reaction in a total of 15 μL mixture (5 PCR product and 10 ul of digestion mixture), incubated at the respective endonuclease temperature (37°C) for a total 3-h duration. The finally digested products were visually tested on 0.8% agarose gel electrophoresis at 150-volts for 35 min, gel images were captured, and polymorphism level was validated among three testing genotypes (P_1_, P_2_, and F_1_), respectively. The finally screened polymorphic markers were effectively genotyped with newly developed F_2_ population, and homozygous and heterozygous allelic bands were coded for mapping of respective QTLs.

### Linkage Mapping, QTL Detection, and Genes Prediction

A genetic linkage map was developed and putative QTLs were detected by using the default parameter settings of the integrated IciMapping (v4.2) tool as followed (Meng et al., 2015). The coded genotypic data of allelic bands of 100-F_2_ mapping population was grouped and anchored over the whole genome chromosomes according to their exact physical positions and maximum likelihood means, using the “rippling” method. The default Chi-squared method was used to determine the segregation ratio of genotypic markers and distorted markers were eliminated at *p*-value of > 0.001. The default kosambi mapping function and recombination frequency value (0.35 rf) were used to estimate the genetic distances, intervals, and positions in centimorgans (cM), respectively.

Quantitative trait loci were detected by using the inclusive composite interval mapping with additive effects (ICIM-ADD) approach using the sliding parameter (1 cm) and permutation testing (1000×) at *p-*value of 0.05 (Amanullah et al., 2021). The significant QTLs were defined as above the threshold level of the default set cut-off logarithm of odd (LOD) score (2.50) and genome-wide type I error at α = 0.05. QTLs with >10% phenotypic variance explained (PVE) and high LODs were named major-effect QTLs, and QTLs with <10% PVE and low LODs were named minor-effect QTLs. Then, all the mapped QTLs were abbreviated as follows; trait name, chromosome number, and QTL number, respectively.

The detected physical positions of flanking CAPS marker sequences were pair-wise aligned over the genomic sequences of re-sequencing data of both parent materials and *de novo* reference genome database of watermelon (97103, v2) (http://cucurbitgenomics.org/), using the web-based application of Integrative Genomics Viewer (IGV, v2.12.2). Then, total candidate genes underlying the adjacent genomic regions were predicted according to their functional annotation of GO database and major SNP mutations within gene coding region were identified, respectively.

### Statistical Data Analysis

The collected quantitative dataset of phenotypic traits was computed on a Microsoft Excel worksheet (v2016) and statistically analyzed, e.g., descriptive statistics of means, standard deviations (SDs), and frequency distributions, by using the default quantitative methodology of statistics software suit (IBM-SPSS, v26.0). The Pearson's correlation coefficients of the observed dataset were analyzed and graphically represented by using the R program (v4.1.2) with its RStudio interface and functional packages “ggplot2” and “corrplot”, respectively.

## Results

### Mapping of Re-sequencing Reads

The mapping statistics of whole-genome re-sequencing reads of two contrasted parent lines of watermelon is shown respectively (Supplementary Table S1). A total of 98.93% (416 Mb) of *de novo* assembled reference genome was covered by re-sequencing reads of LSW-177 (P_1_). In sum, 13,347,1440 reads were QC-passed and properly paired, and 271,440 reads were as supplementary reads, from which 13,204,4185 reads were efficiently mapped, 12 million (12,200,6330) reads (about 91.60%) were properly paired (map Q≧5), as followed by other types of sequencing reads.

A total of 98.94% (416.50 Mb) of the reference genome was covered by the re-sequencing reads of ZXG-1555 (P_2_). In sum, 69,708,578 were QC-passed reads and paired-end sequencing reads, and 1,831,128 were supplementary reads. About 68,971,776 reads (< than total) were successfully mapped, and 60,649,000 reads (about 89.35%) were properly paired (map Q≧5), as followed by another type of re-sequencing reads. The whole-genome re-sequenced reads of both parent lines suggested a very close relationship with the reference genome (V2) of watermelon (97103). Overall, the re-sequencing of both parent lines exhibited that P_1_ parent reads were mapped higher than P_2_ parent reads, and these reads were further used for bioinformatics analysis and molecular experimentation.

### Density of SNP Variants

**Figure 2:**
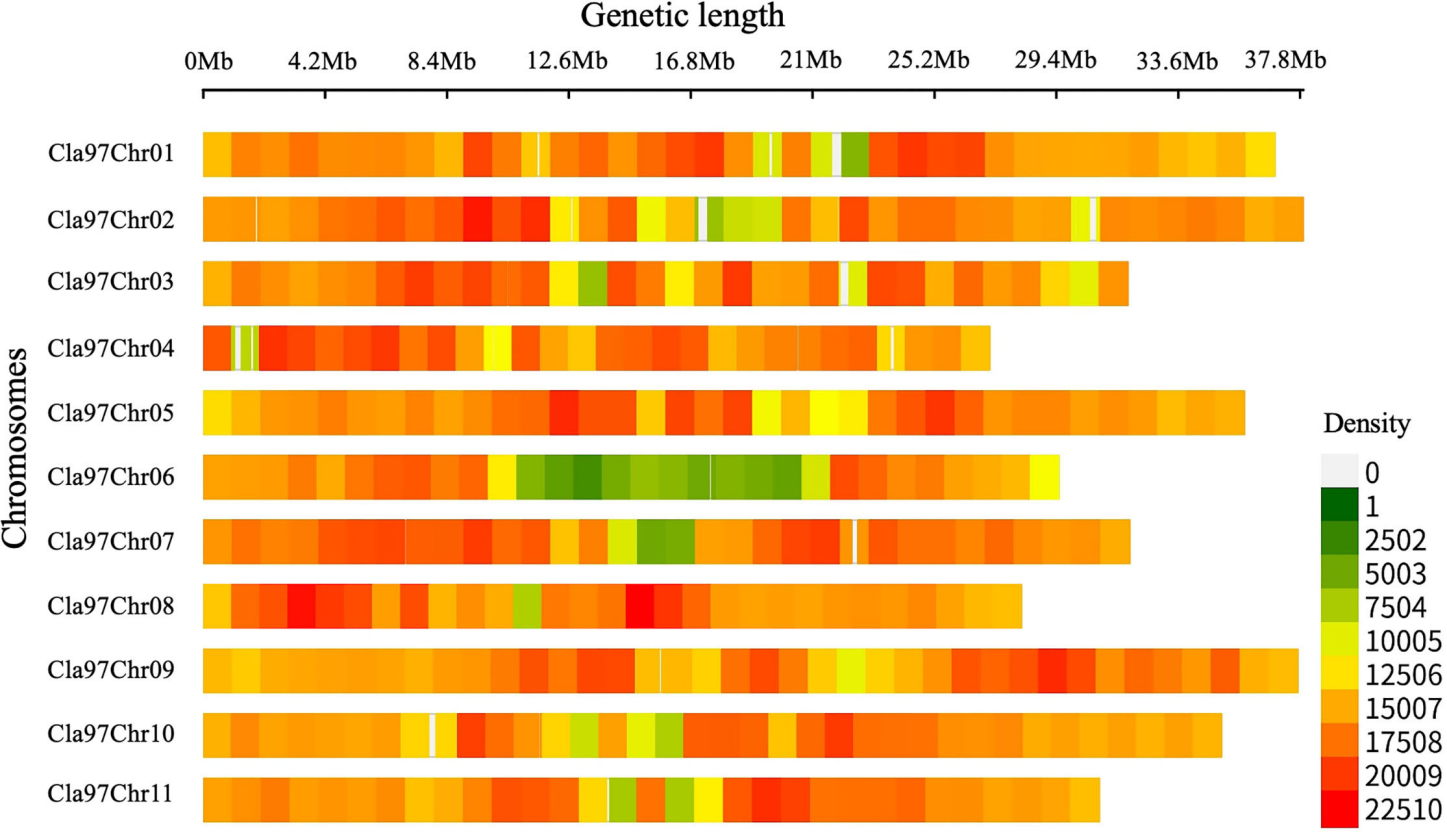
Total SNP variants detected within a 1-Mb window-sized chromosomal region of re-sequenced parent lines of watermelon.

Whole-genome detected SNP variants were pre-filtered and then characterized respectively (Figure 2, Supplementary Table S2), by using the bioinformatics analysis and aligning the mapped re-sequenced reads of two contrasted parent lines over the chromosomal genetic length of reference genome of watermelon (97103, v2). A total of 363,083,641 bp of genetic length was covered by whole-genome chromosomes (Cla97Chr01 to Cla97Chr11), among which Cla97Chr02 exhibited the maximum genomic coverage of 37.92 Mb (37,915,939 bp) length, Cla97Chr04 exhibited a minimum coverage of 27.11 Mb (27,110,815 bp) length, and the remaining chromosomes exhibited the average length of genomic coverage.

Further, SNP variants density was checked within 1-Mb window-sized chromosomal regions of whole-genome (Cla97Chr01 to Cla97Chr11). A total of 49,89,869 SNP variants with an average rate of 72 variants/kb were observed, and these variants were randomly localized over the whole genome chromosomal length. The majority of SNP variants (532,615) were noticed within the genetic length (35.93 Mb) of Cla97Chr09 with an average of 70 variants/kb. The least number of SNP variants (326,114) were exhibited within the genetic length (29.50 Mb) of Cla97Chr06 with an average rate of 90 variants/kb. Regarding the total average variant rate per kb, a minimum average of 68 variants/kb was observed within the genetic length of Cla97Chr08 and a maximum average of 72 variants/kb was observed in Cla97Chr06.

### Characterization of SNP Variants

**Figure 3:**
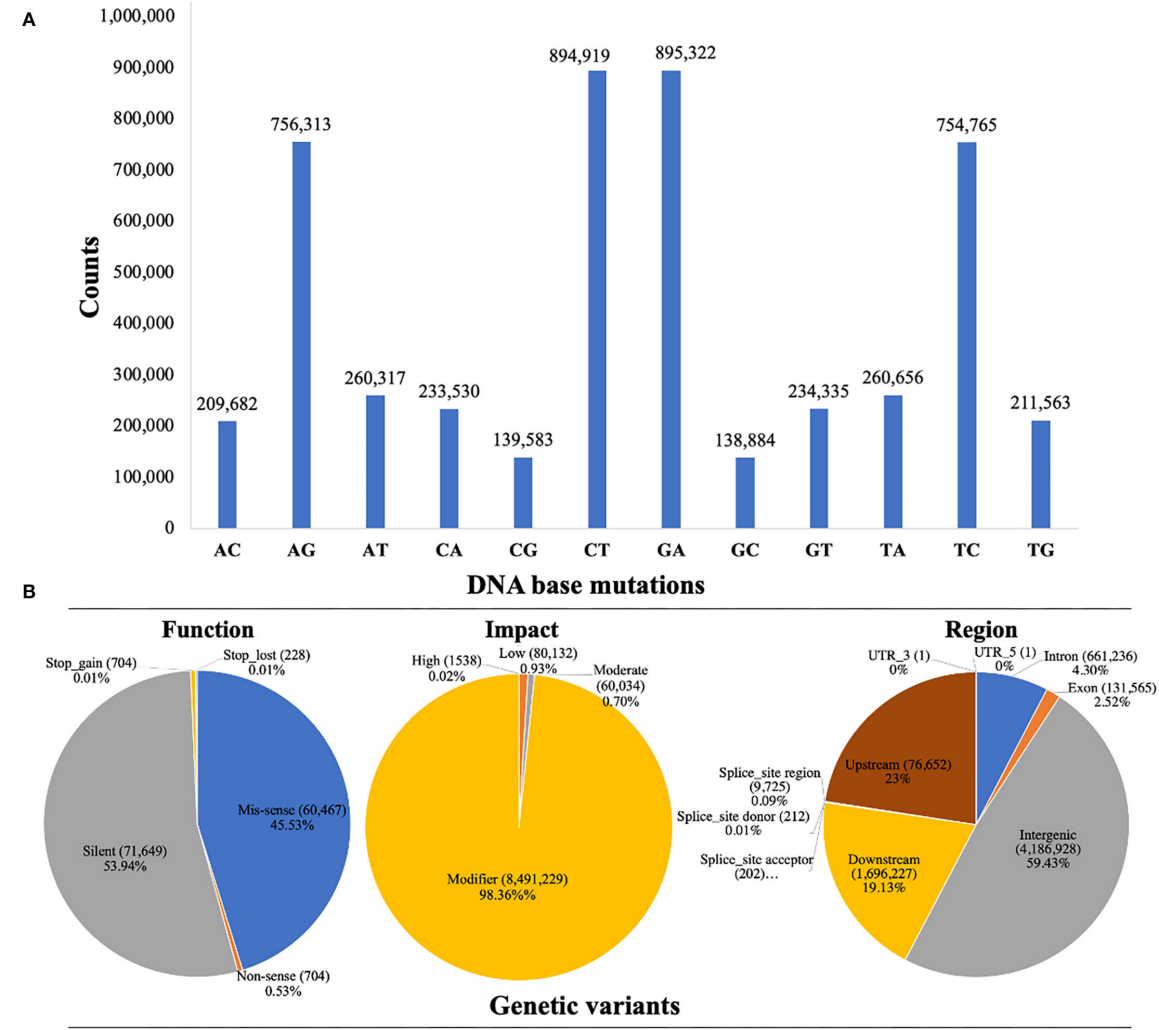
Whole genome characterization and annotation of detected SNP variants based on re-sequencing data of watermelon parent lines. **(A)** Frequency distribution of SNP variants with transitions and transversions. **(B)** SNP variants proportion by their classification.

Whole-genome detected SNP variants were well-characterized into transversion-type and transition-type variants as shown in Figure 3A. A total of 99.96% transversions-type (1,688,505 counts) and 99.97% transitions-type (3,301,319 counts) variants were detected across the whole genome re-sequenced reads, respectively. Among the transversion variants, the maximum DNA-base mutations were noticed for 26.24% of AC (209,682 counts) → CA (233,530 counts), 26.40% of TG (211,563 counts) → GT (234,335), and 30.85% of AT (260,317 counts) → TA (260,656 counts), and minimum substitution mutations were noticed for 16.48% of CG (139,583 counts) → GC (138,884 counts), respectively. Among the transition variants, 50% of AG (756,313 counts) → GA (895,322 counts) substitution mutations were observed, which was lower as compared to the 49.97% of CT (849,919 counts) → TC (754,765 counts). However, the ratio of total transitions-type to total transitions-type (TSs/TVs) was 1.95%.

### Annotation of SNP Variants

The annotation of SNP variants was performed to assess the proportions of potential genetic variants by their possible function, impact, and region, along with counts and percentages (%) across the re-sequenced watermelon parent lines (Figure 3B). The highest rate of genetic variants (4,186,928 counts) was identified in 48.50% of intergenic_region, 1,946,227 variants were identified in 22.54% of the upstream_region, 1,696,837 variants were identified in the 19.65% of the downstream_region, and 661,236 variants were identified in 7.6% of the intron_region. However, the lowest variants (131,565 counts) were present in 1.52% of the intron_region, respectively.

The SNP variant effects were organized into 4 types, e.g., “high_effect type”, “moderate_effect type”, “low_effect type”, and “modifier_type”. A total of 98.36% of SNP variants were identified with modifier impact in the intron or exon region, 1,538 variants (0.02%) were with high_effects, 80,132 variants (0.93%) were with low_effects, and 60,034 variants (0.70%) were with low_effects. A total of three SNP variant types were identified according to their functional categories. A total of 45.53% of missense variants (60,467 counts) were identified, which switched one amino acid into chain form by connecting it with others. There was a total of 0.53% non-sense SNP variants (704 counts), which caused a shorter chain due to the early stop codon. However, 53.94% of silent mutations (caused by a single-base substitution mutation) were detected with 71,649 counts, where the encoding function of proteins was never altered. The missense to silence class ratio was about 0.84.

### Development of Polymorphic Markers

Whole-genome re-sequencing reads (>25× depth coverage) of two-parent lines were aligned over the reference genome (v2) of watermelon (97103), respectively. A total of 1,82,949 CAPS loci pairs were excavated by filtering the existence and non-existence sites of suitable endonucleases in each SNP location (Supplementary Table S1). A minimum of 11,688 CAPS loci pairs were positioned in Cla97Chr08 and a maximum of 20,830 CAPS loci pairs were noticed in Cla97Chr09. Overall, 5-endonucleases (*Msp* I, *Hin*d II, *Eco*R I, *Bsa*H I, *Pst* I, *Bam*H I) sites of major CAPS loci were perfectly matched and these were selected for the design of whole-genome markers according to experimental conceptualization.

**Figure 4:**
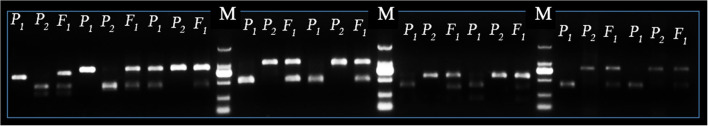
Identification of SNP-CAPS markers polymorphism between female parent (LSW-177 as P_1_), male parent (ZXG-1555 as P_2_), and their F_1_ progeny. The letter “M” is the marker (DL2000) ladder, respectively.

**Figure 5:**
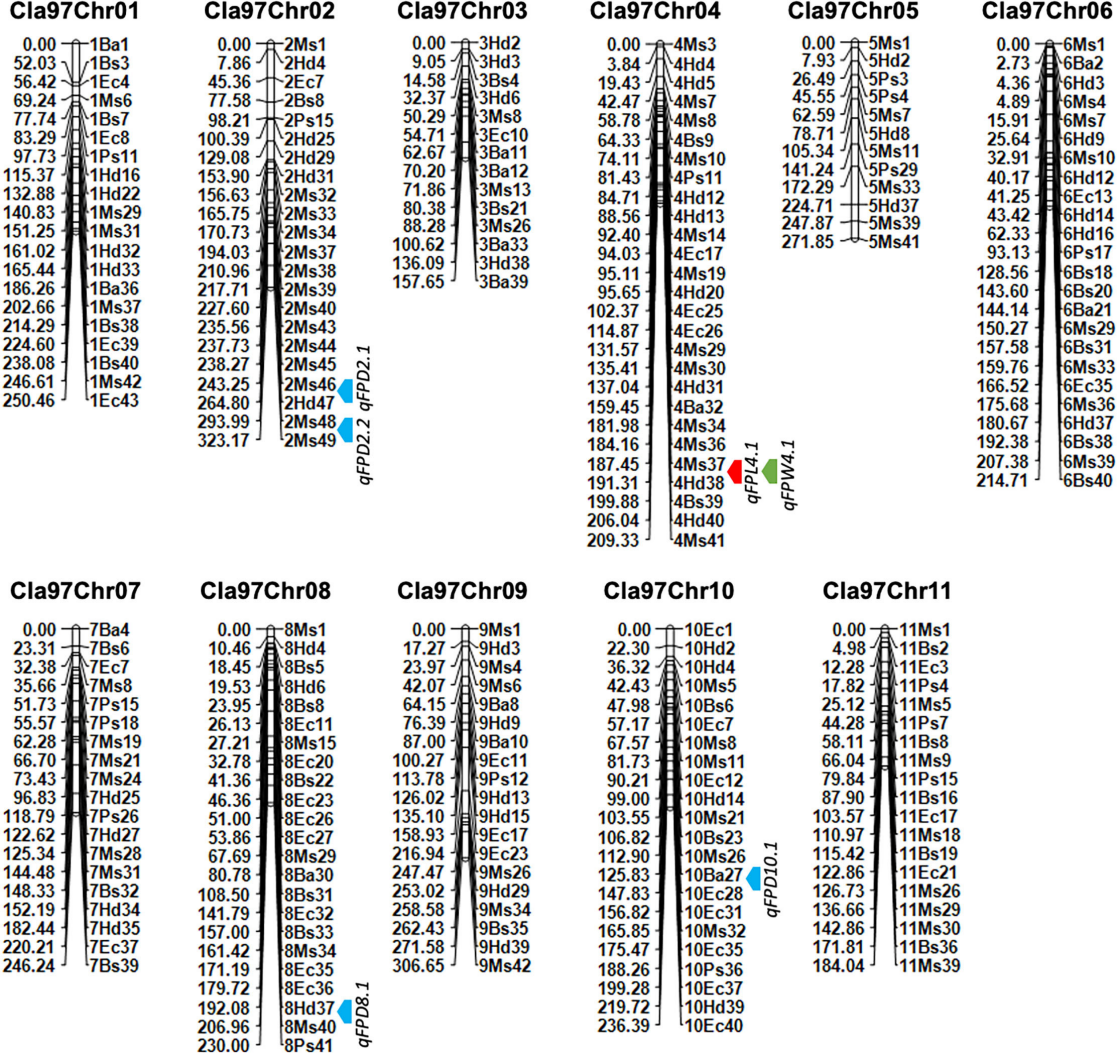
A developed genetic map of watermelon based on genotyping of novel 221 SNP-CAPS markers within F_2_ mapping individuals. The bold color arrows (red, blue, and green) indicate the mapped QTL regions. Marker's positions are aligned on the right side and their genetic positions are aligned on the left side of each chromosome, respectively.

In sum, 476 pairs of randomly selected CAPS sequences were used for primer designing and further identification of codominant polymorphisms among the DNA of P_1_, P_2_, and F_1_ progenies. The amplified fragmented PCR products and enzyme digested products were cleaved at 0.8% agarose gel electrophoresis. A total of 221 pairs of CAPS primers exhibited the properly digested products within exact base pair (bp) lanes of polymorphic bands and validated the 46.42% cleaved codominant polymorphisms. The remaining 255 CAPS pairs produced the same band patterns (did not show polymorphism) or failed to show proper enzyme digestion, so we excluded those primer pairs. Finally, the enzyme digested products and polymorphic bands of P_1_, P_2_, and F_1_ genotypes were visually observed in different bp lanes (Figure 4). We noticed that all the F_2_ genotyped polymorphic markers were located on each chromosome by their proper physical positions (bp), fragment product lengths, and endonuclease sites (Figure 5).

### Analysis of Developed Linkage Map

A genetic linkage map was developed by using the coded genotypic data of 221 pairs of novel codominant CAPS markers within 100-F_2_ mapping population developed from two parent materials of watermelon (Figure 5, Table 1). The genotypic percent for allele frequency was observed as AA: 24.7%, BB: 24.4%, and AB: 50.7%, respectively. The orders of codominant markers were randomly distributed across the whole-genome linkage map based on their physical positions (bp) and screened fragment lengths. Out of 221 pairs, a total of 200 CAPS markers (90.49%) exhibited the exact ratio of Mendel's segregation (1:2:1) at the *p*-value of > 0.05, except for the 21 pairs of CAPS markers (9.05%), which indicated the distorted segregation ratio.

**Table 1:** Summary of markers distributions across the developed genetic linkage map of watermelon.

**Chromosomes**	**Genetic length (cM)**	**Polymorphic markers #**	**Average length (cM)**	**Spearman's correlation**
Cla97Chr01	250.46	20	12.52	0.987
Cla97Chr02	323.17	22	14.67	0.956
Cla97Chr03	157.65	14	11.26	0.945
Cla97Chr04	209.33	27	7.75	0.968
Cla97Chr05	271.85	12	22.65	0.968
Cla97Chr06	214.71	24	8.94	0.962
Cla97Chr07	246.24	19	13.00	0.954
Cla97Chr08	230.00	23	10.00	0.926
Cla97Chr09	306.65	19	16.13	0.975
Cla97Chr10	236.39	22	10.74	0.987
Cla97Chr11	184.04	19	9.69	0.987
**Total**	**2,630.49**	**221**	**11.90**	**0.965**

*The bold values denote the total calculation of each column data, and # symbol denotes the total numbers of CAPS markers on each chromosome*.

The final genotyped markers were exactly positioned with goodness-of-fit over the whole genome chromosomes (Cla97Chr01 to Cla97Chr11). The developed linkage map covered a total genetic length of 2,630.49 cM with an average of 11.90 cM. An average genetic length ranged from a minimum of 157.65 cM (Cla97Chr03) to a maximum of 323.17 cM (Cla97Chr08), respectively. The marker numbers were also varied and ranged from 14 (Cla97Chr03) to 27 (Cla97Chr04) with 209.33 cM length and 7.75 average; however, most of the chromosomes contained a maximum and a normal number of markers (>20 markers). The proper genetic positions of the markers facilitated the possible development of a quality genetic linkage map and fine QTL mapping.

The collinearity analysis was also performed between whole-genome genotypic CAPS markers information and the developed physical and genetic map (Supplementary Figure 2). The scatter plot of the collinearity test also depicted positive and linear regression (*r*2 = 0.92 to 0.98) values, because the straight curve illustrated an appropriate, increased, and fitting trend of the marker's physical positions (bp) and genetic positions (cM) over the whole-genome chromosomes. Three chromosomes (Cla97Chr04, Cla97Chr06, and Cla97Chr08) contained a few markers with partial segregation that were somewhat deviated from their best fit line, and an inversion in the collinearity curve also represented the extended genetic gaps between the flanking positions of adjacent markers. We suggest that further re-sequencing data based on high-density markers should overcome this problem.

### Analysis of Phenotypic Variability

**Figure 6:**
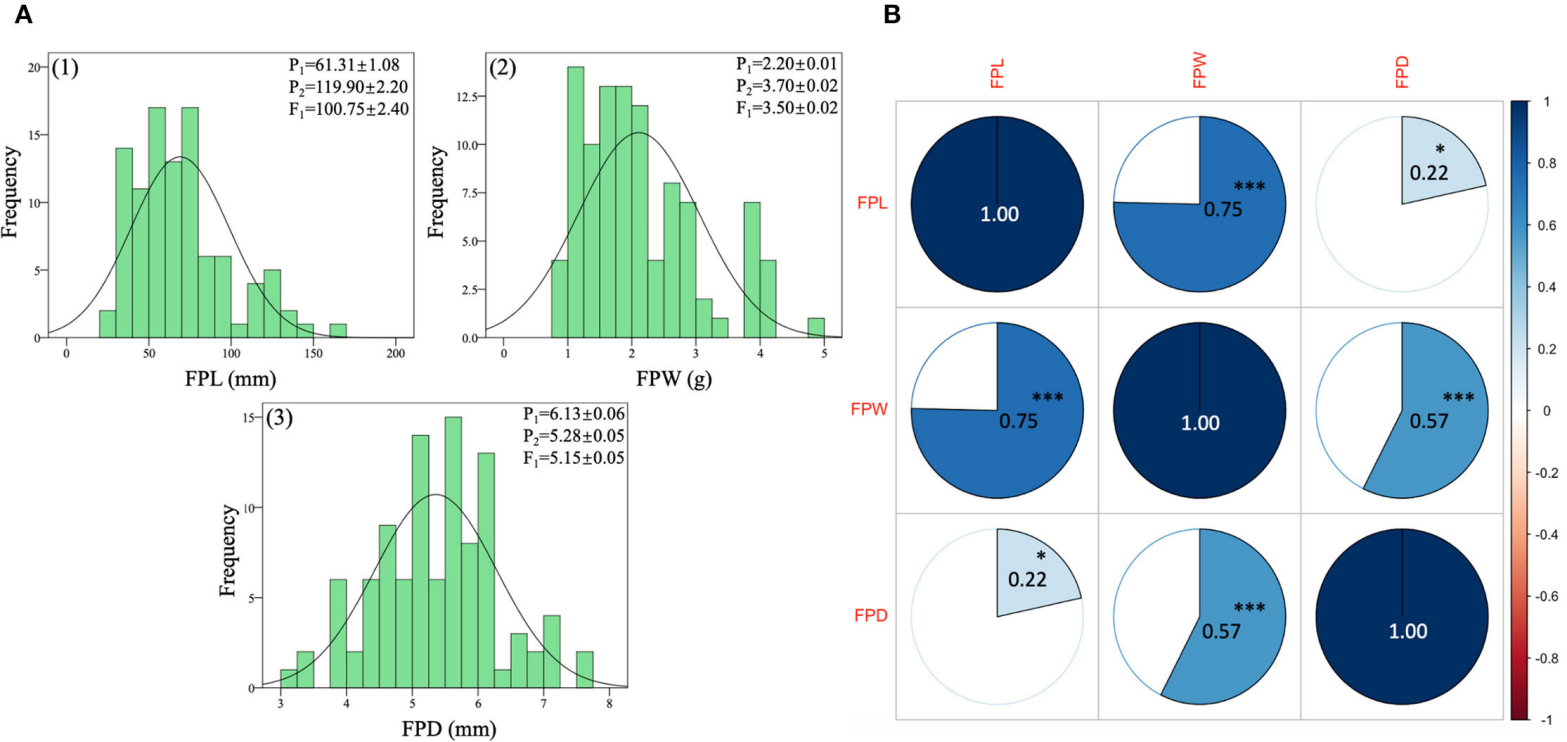
Phenotypic analysis of FP traits of contrasted parent lines, F_1_ progeny, and derived F_2:3_ mapping individuals of watermelon. **(A)** Histogram of frequency distributions of measured traits. **(B)** Pearson's correlation coefficient values of measured traits. The color pie charts and scale bar denote suggestive colinearity at both levels (^***^p < 0.01 and ^*^p < 0.05).

The quantitative dataset of FP traits was collected from two contrasted parent lines (LSW-177 and ZXG-155), F_1_ progeny, and respective F_2:3_ mapping population. The statistical data analysis depicted normal frequency distribution, transgressive segregation, and highly significant correlation coefficients between the observed traits as shown respectively (Figure 6, Supplementary Table S3).

### Cytological Observation of Pedicels

We checked the longitudinal sections of paraffin-embedded FP samples at 40× magnification (0.1 mm scale) (Figure 1B). There were significant differences in the centered pith cell numbers of FPs of P_1_ (LSW-177), P_2_ (ZXG-1555), and F_1_ samples, and calculated cell numbers of P_1_ were 36, P_2_ were 92, and F_1_ were 90, respectively. The optical cell size was also different between the observed pedicel samples, implying that the longer pedicel phenotype of P_2_ parent and F_1_ progeny is mainly responsible for the greater pedicel weight variation in F_2:3_ mapping population.

### Fruit Pedicel Length (FPL, mm)

The FPL (mm) values of both parent lines and their F_1_ exhibited different means due to the major variability in pedicel lengths (long and short pedicel). The values of P_1_, P_2_, and F_1_ progeny were 61.31 ± 1.08 mm, 119.90 ± 2.20 mm, and 100.75 ± 2.00 mm, respectively. The F_1_ pedicel length showed higher mean values (mm) than the P_1_ parent values but very close to the mean values of P_2_ parent. In the F_2:3_ population, the FPL mean value was 16.52 ± 3.91 mm, and the range varied from 9 mm to 52 mm, respectively. Further, a standard distribution frequency and a transgressive segregation ratio were noticed in phenotypic analysis of pedicel traits (Figure 6A-1). The observed phenotypic characteristic showed a complete type of dominance effect, inherited from the FP length trait of P_2_ parent.

### Fruit Pedicel Weight (FPW, g)

The FPW (g) values of both parent lines and their F_1_ also exhibited distinct means (Figure 6A-2) due to the significant variability in pedicel weight morphology (more and less weight). For P_1_, P_2_, and F_1_ progeny, the averaged values were 2.20 ± 0.01 g, 3.70 ± 0.02 g, and 3.60 ± 0.02 g, separately. The values of FPW of F_1_ progeny were also higher than female P_1_ parent values and tightly related with the P_2_ parent values. The overall mean of FP weight of the F_2_ population was 2.11 ± 0.94 (g), and variability ranged from the lowest value of 1 g to the highest value of 5 g, with a uniform frequency distribution. However, the male parent line (P_2_) seemed to show a major inheritance effect with more FP weight.

### Fruit Pedicel Diameter (FPD, mm)

The phenotypic values of FP diameter (FPD) of contrasted watermelon parent lines were also distinctive. For P_1_, P_2_, and F_1_ progeny, the mean values were 6.13 ± 0.06 mm, 5.28 ± 0.05 mm, and 5.15 ± 0.05 mm, respectively. The values of FPD of F_1_ progeny depicted the close genetic inheritance of pedicel diameter received by the P_2_ parent (with less diameter but a connection with more pedicel weight). In the F_2_ mapping population, the mean value of FPD was 5.36 ± 0.93 mm and range were from 3 mm to 8 mm, respectively (Figure 6A-3). A uniform distribution of FP weight frequency and variability was in line with differential QTL effects on different chromosomes. Overall, a transgressive frequency was observed for the collected datasets of F_2:3_ mapping population, suggesting the important quantitative nature of FP traits.

### Phenotypic Correlation

The correlation matrix indicated a good connection among the FP-associated traits (Figure 6B). A positive and highly significant correlation coefficients (0.75^***^, 0.57^***^, 0.22^*^) were observed among the FP traits (FPL, FPW, FPD) at both levels of ^***^p- value of < 0.01 and ^*^p-value of < 0.05, respectively. It was noticed that a close relationship between the entire variability of FP traits was present in the F_2:3_ mapping population, which suggested a clear quantitative genetic nature, inherited from both parents and F_1_ progeny. The well-connection between observed variables also proposes that elongated FPs with a moderate diameter have more pedicel weight than those of short-length pedicels with less weight.

### QTL Mapping

**Figure 7:**
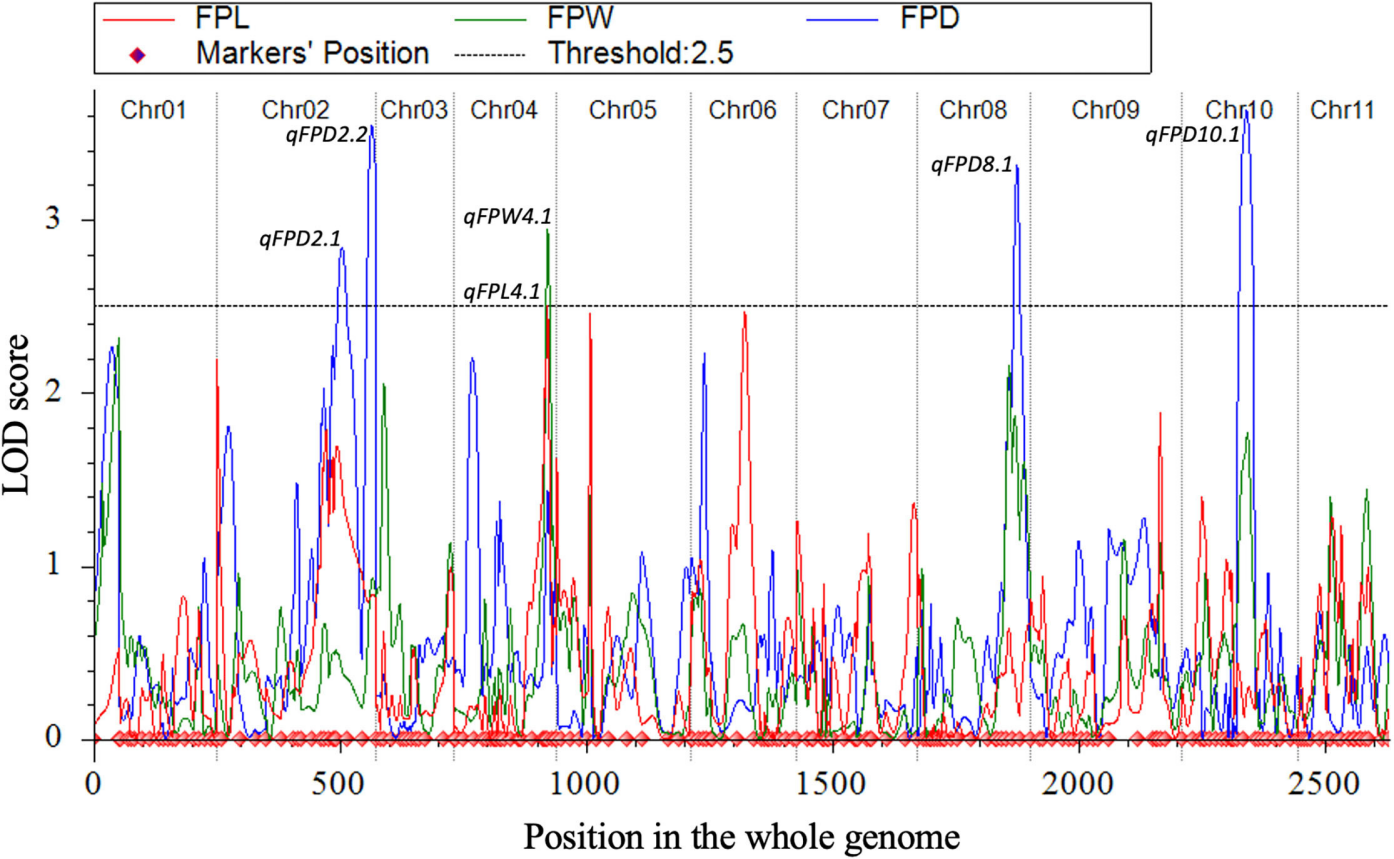
All LOD score profiles of mapped QTLs for FP traits and their genetic positions in the whole-genome.

Quantitative trait loci were mapped by combining the coded genotypic data of codominant markers and the means of quantitative datasets of FPL, FPW, and FPD of 100-F_2:3_ mapping individuals. Overall, 6 QTLs (5 major-effect and 1 minor-effect) were mapped. Among them, 1 major-effect QTL of FPL, 1 major-effect QTL of FPW, and 4 QTLs (3 major-effect and 1 minor-effect) of FPD were localized between the flanking genetic regions of codominant CAPS markers, respectively. The details of genetic effects of mapped QTLs, e.g., detected LOD scores, additive (Add) effects, dominance (Dom) effects, phenotypic variance explained (PVE%), flanking markers (left-right), their physical positions (bp), and genetic positions (cM) are shown respectively (Figures 5, 7, Table 2).

**Table 2:** Genetic effects of identified QTLs controlling watermelon FP traits.

**QTLs**	**Chromosome IDs**	**Genetic** **positions (cM)**	**Left-Right markers**	**Physical positions (bp)**	**LOD** **scores**	**PVE (%)**	**Add** **effects**	**Dom effects**
*qFPD2.1*	Cla97Chr02	254	2Ms46-2Hd47	35039739–35988127	2.84	8.64	0.33	0.38
*qFPD2.2*	Cla97Chr02	315	2Ms48-2Ms49	36931835–37879344	3.54	12.76	0.33	0.64
*qFPL4.1*	Cla97Chr04	191	4Ms37-4Hd38	24392485–25067584	2.52	10.46	13.45	−8.25
*qFPW4.1*	Cla97Chr04	191	4Ms37-4Hd38	24392485–25067584	2.94	13.60	0.43	−0.48
*qFPD8.1*	Cla97Chr08	202	8Hd37-8Ms40	25356537–26107916	3.31	10.99	−0.35	−0.56
*qFPD10.1*	Cla97Chr10	132	10Ba27-10Ec28	22813961–23673195	3.63	13.13	0.54	−0.11

### QTLs of FPL

For the FPL, a single major-effect QTL (*qFPL4.1*) was mapped near the bottom end of chromosome (Cla97Chr04). The LOD score of 2.52, Add effect of 13.45, and Dom effect of −8.25 were observed. This QTL explained a total of 10.46% PVE for individual trait effect, and detected positive Add effect significantly described the main genetic contribution of elongated FPs in the derived mapping population. Regarding the genetic location (cM), *qFPL4.1* was positioned at 191 cM between the flanking regions of two left-right codominant CAPS markers “4Ms37 and 4Hd38”. These two markers were positioned at 187.50 cM and 195.50 cM, and a short genetic interval of 8 cM was noticed between those genetic regions. The physical position (24392485-25067584 bp) of two flanking markers exhibited a total of 675.10 kb genetic interval for detected QTL (*qFPL4.1*), respectively.

### QTLs of FPW

Interestingly, a single major-effect QTL (*qFPW4.1*) was found to be co-localized with a major-effect QTL of FPL (*qFPL4.1*) near the bottom end of chromosome (Cla97Chr04), by sharing a common genetic region. For the *qFPW4.1*, LOD score of 2.94, Add effect of 0.43, and Dom effect of −0.48 were observed, and this QTL explained a total of 13.60% PVE with individual trait effect, and highly positive Add effect significantly described its major contribution for variation in FPs with more weight in derived mapping population. Regarding the genetic location (cM), *qFPW4.1* existed at 191 cM between the flanking sections of two left-right codominant CAPS markers “4Ms37-4Hd38” positioned at 186.50 cM and 195.50 cM, and a short genetic interval of 7.5 cM was noticed between them. The same physical position (24392485-25067584 bp) and genetic interval of 675.10 kb were noticed for both QTLs (*qFPL4.1* and *qFPW4.1*) between the same flanking markers. Further, these two QTLs mutually explained a total of 24.06% PVE and mutually indicated the significant contribution of genetic inheritance of lengthy and weighted FPs in the derived F_2:3_ mapping population, which might be mainly transferred by P_2_ parent.

### QTLs of FPD

A total of 4 QTLs (3 major-effects and 1 minor-effect) of FPD (*qFPD2.1, qFPD2.2, qFPD8.1*, and *qFPD10.1*) were mapped on three different chromosomes (Cla97Chr02, Cla97Chr08, and Cla97Chr10), showing an unlinked and diverged multi-genic architecture in the watermelon genome, respectively. A major-effect QTL (*qFPD2.2*) was identified just below the flanking position of nearly positioned minor-effect QTL (*qFPL2.1*) at the bottom region of chromosome (Cla97Chr02), and detected with an LOD score of 3.54, Add effect of 0.33, Dom effect of 0.63, and 12.76% of PVE. This QTL (*qFPL2.2*) was genetically positioned at 315 cM between two flanking left-right codominant CAPS markers “2Ms48-2Ms49” pinpointed at 304.50 cM and 323 cM position, with a genomic interval of 18 cM, respectively. The physical position (36931835-37879344 bp) between two markers exhibited the 947.51 kb genetic distance for *qFPD2.2*. Another single minor-effect QTL (*qFPD2.1*) was similarly dentified at the bottom region of chromosome (Cla97Chr02), and detected with an LOD score of 2.84, Add effect of 0.33, Dom effect of 0.38, and 8.64% of PVE. This QTL (*qFPL2.1*) was positioned at 254 cM between two flanking left-right codominant CAPS markers “2Ms46-2Hd47” pinpointed at 238.50 cM and 280 cM, having a genomic interval of 42 cM, respectively. The physical position (35039739-35988127 bp) of these two markers exhibited 948.39 kb genetic interval for *qFPD2.1*.

Another major-effect QTL (*qFPD8.1*) was discovered separately at the end of the chromosomal region of Cla97Chr08, and detected with an LOD score of 3.31, Add effect of −0.35, Dom effect of −0.56, and 10.99% of PVE. This QTL was positioned at 202 cM between left-right flanking codominant CAPS markers “8Ms37-8Ms40” pinpointed at 193.50 cM and 211.50 cM, with genomic interval of 18 cM, respectively. The physical position (25356537-26107916 bp) of these two markers exhibited a total of 751.38 kb genetic distance for *qFPD8.1*. On Cla97Chr10, a separate major-effect QTL (*qFPD10.1*) was also identified with a detected LOD score of 3.63, Add effect of.54, Dom effect of −0.11, and 13.13% of PVE. Regarding the genetic position, *qFPL10.1* was positioned at 132 cM between two left-right flanking codominant CAPS markers “10Ba27-10Ba28” positioned at 116.50 cM and 145.50 cM, exhibiting a genetic interval of 29 cM between them, respectively. The physical position (22813961-23673195 bp) of these two markers revealed total 859.24 kb genetic distance for *qFPD10.1*. However, a total of 45.52% of mutually explained PVE was noticed for all mapped QTLs of FPD. One minor-effect and two major-effect QTLs (*qFPD2.1, qFPD2.2*, and *qFPD10.1*) demonstrated the positive Add effects, predicted by their main genetic inheritance for increased pedicel diameter; however, another major-effect QTL (*qFPD8.1*) explained the negative additive effect by showing the inheritance of a pedicel with less diameter and weight.

### Prediction of Putative Genes and Annotation

**Figure 8:**
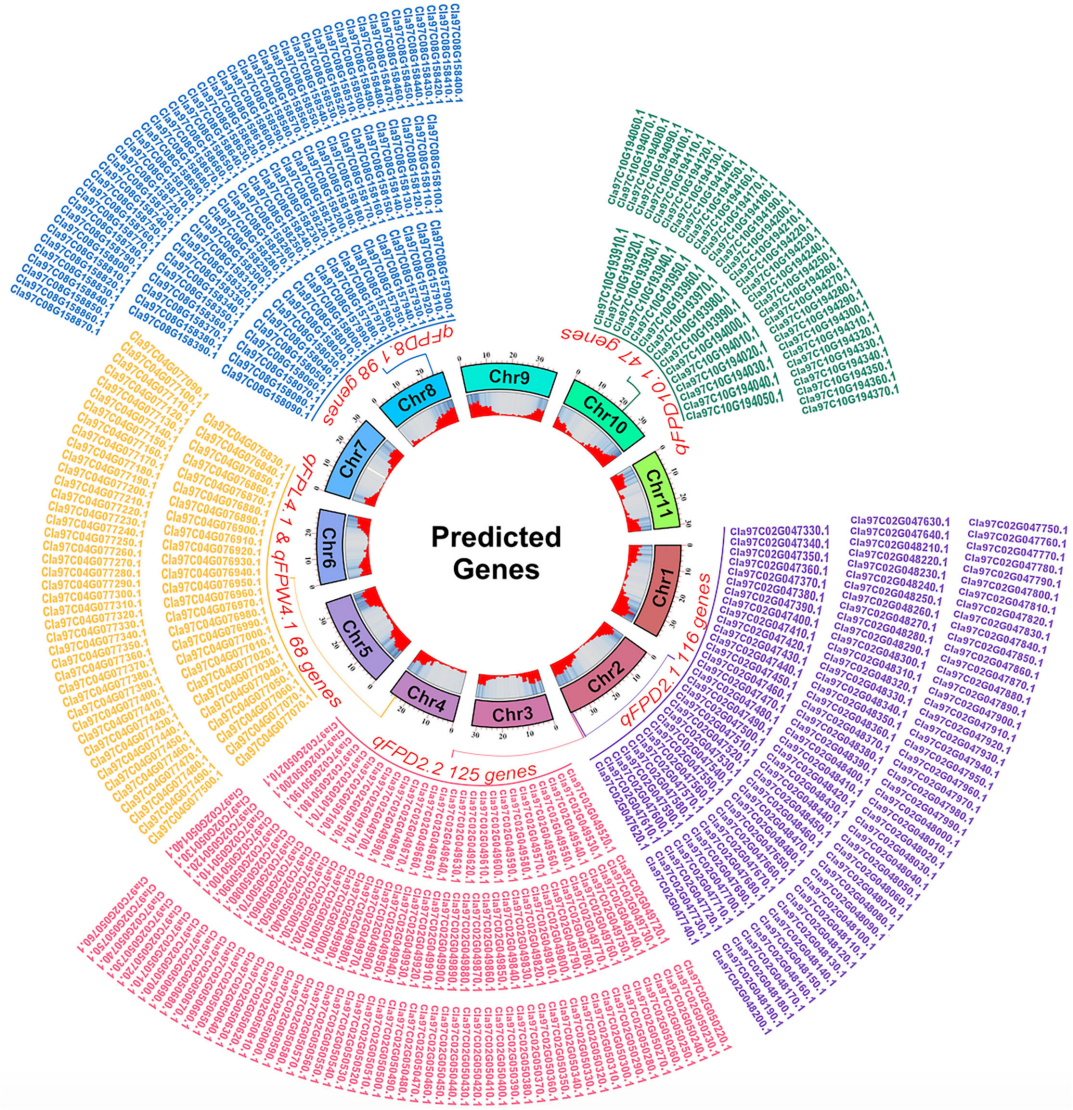
A circle diagram of putative genes predicted in flanking QTL regions controlling FP traits in watermelon.

The pair-wise alignment of physical sequences of delimited flanking QTLs and reference genome database of watermelon (97103, v2) revealed a total of 454 putative genes related with FPL, FPW, and FPD were predicted, and presented with different generic gradients of circle diagram (Figure 8), and detailed functional GO annotation of defined genes and detected SNPs in coding region is also mentioned respectively (Supplementary Tables S4–S8).

For the FPD, a total of 386 genes were identified in the 4 QTL regions, e.g., 116 genes in the 948.39 kb region of *qFPD2.1*, 125 genes in the 947.51 kb region of *qFPD2.2*, 98 genes in the 751.38 kb region of *qFPD8.1*, and 47 genes were identified in the 859.24 kb region of *qFPD10.1*), respectively. The genes annotation analysis described that most of the genes have known functions but very few of them exhibited unknown functions. All the annotated genes were also categorized in the molecular function, cellular components, and biological processes, respectively. However, some genes exhibited more explicit description, e.g., *Cla97C02G047510* (Auxin-responsive family protein), *Cla97C02G048250* (ABSCISIC ACID-INSENSITIVE 5-like protein 1), *Cla97C02G048310* [Late embryogenesis abundant (LEA) hydroxyproline-rich glycoprotein family], *Cla97C02G048480* [Late embryogenesis abundant (LEA) hydroxyproline-rich glycoprotein family], *Cla97C02G050460* and *Cla97C02G050490* [Late embryogenesis abundant (LEA) hydroxyproline-rich glycoprotein family], *Cla97C02G050510* (GDSL esterase/lipase family), *Cla97C08G157940* (Cytokinin riboside 5'-monophosphate phosphoribohydrolase), *Cla97C08G158260* (Cytokinin dehydrogenase 5), *Cla97C10G193930* (Late embryogenesis abundant protein 3L-1), *Cla97C10G193940* (Late embryogenesis abundant protein 5), *Cla97C10G194030* (Ethylene responsive transcription factor 2b), and *Cla97C10G194120* (Long cell-linked locus protein).

For the FPL and width, a common genetic position was shared by two co-QTLs (*qFPL4.1* and *qFPW4.1*) which exhibited a total of 68 genes in 675.10 kb region, respectively. Some genes similarly exhibited more explicit descriptions, e.g., *Cla97C04G076840* (germin-like protein subfamily 3 member 2), *Cla97C04G077140* and *Cla97C04G077150* (IAA-amino acid hydrolase ILR1), and *Cla97C04G077450* (Auxin efflux carrier component). Overall, non-synonymous SNPs (changing the codon to one that codes for a different amino acid) were found in the majority of annotated genes; however, a greater number of gene SNPs were noticed in the CDS coding regions of ZXG-1555 (P_2_) as compared to LSW-1555 (P_1_).

## Discussion

Over the last few decades, different genetic mapping strategies and DNA markers [randomly amplified polymorphic DNA (RAPDs), simple Sequence repeats (SSRs), restriction fragment length polymorphisms (RFLPs), amplified fragment length polymorphisms (AFLPs), expressed sequence tags (ESTs), SNPs, and CAPSs] have demonstrated their effectiveness for identifying the potential genetic loci affecting multiple crop traits by using different types of mapping populations (Amanullah et al., 2018). The major limiting factor in developing the efficient genetic markers and their experimental genotyping for linkage map construction is cost-effectiveness (Pereira et al., 2018; Amanullah et al., 2020). However, the size of mapping populations and the density of whole-genome markers are similar concerns for perfect QTL mapping (Amanullah et al., 2021).

Fruit pedicels are important determinants of premium fruit quality and market worth. In the current study, newly derived whole-genome SNP-derived CAPS markers identified a total of 6 potential QTLs affecting FP traits (length, width, and thickness), over the dissimilar genetic locations of whole-genome chromosomes (Figure 5, Table 2). Regarding the FP length and weight, two tightly overlapped and major-effects QTLs (*qFPL4.1* and *qFPW4.1*) shared a common genetic position on Cla97Chr04 and disclosed the goodness-of-fit association between markers. A total of 24.06% PVE (10.46 and 13.60%, separately) and their positive additive effects (13.45 and 0.43) similarly indicated that pedicel length and weight might be regulated by either a single major gene or by minor polygenic architectures. For the FPD, a total of four QTLs (*qFPD2.1, qFPD2.2, qFPD8.1*, and *qFPD10.1*) were similarly mapped across Cla97Chr02, Cla97Chr08, and Cla97Chr10. Overall, 45.52% PVE (8.64, 12.76, 10.99, and 13.13% separate) of these four QTLs indicated the major genetic contribution for inheritance of pedicels with both of less diameter and weight in F_2:3_ population, and similarly exhibited diverse genetic effects in watermelon genome.

To date, no prior QTL study on watermelon FP traits has been reported. However, a few QTL studies in cucumber, pepper, and melon have been performed in recent years. It was stated in accordance to our study that genetic inheritance of cucumber FNL is controlled by single major gene or by minor polygenes with additive-dominant effects (Ma et al., 2010), and one major-effect QTL “*qfpl6.1*” also showed undefined genetic association against the tested recombinants derived from backcross and biparental generations under the distinct localities (Song et al., 2016). A fine mapped region “*CsFnl7.1*” revealed that cucumber FNL is controlled by a recessive gene, encoding late embryogenesis abundant protein which produce upregulated expressions (Xu et al., 2020). In pepper, 200–250 kb region of *CapUp12.1* defined the regulation of erect FP orientation (*CapUp*), and aco-segregation of traits was observed in F_2_ genotyped population (Solomon et al., 2021). BSA-seq analysis also revealed that major genetic region “*CmFpl3.1*” of 89 kb interval on chromosome 03 is controlling the muskmelon FPL variation by exhibiting good genotypic association (Cui et al., 2022). These genetic mapping studies are in accordance with our results, where different QTLs for pedicel diameter and co-QTLs of length and weight were mapped and showed good genotypic association for pedicel variations, controlled by distinct sequential cell proliferation, cell division, and cell elongation, respectively.

Further, a uniform and transgressive index in this study evidently exhibited the significant variations in FP traits of F_2:3_ population (Figure 6A). A phenotypic dataset analysis similarly exposed highly positive and significant correlations between pedicel traits, respectively (Figure 6B). However, the high correlation and co-localized QTLs (*qFPL 4.1* and *qFPW4.1*) denoted that pedicel length and weight might be shared the same genetic factors or few major and minor polygenic phenomenon. In accordance to our result, it was similarly stated that the FP morphology has a connection with each other since a single change in one trait may totally or partially influence the other trait (Solomon et al., 2021). However, marked QTL effects for hereditary traits frequently diverged in differential genetic backgrounds and environmental localities (Lecompte et al., 2004; Grewal et al., 2010; Palomares-Rius et al., 2016; Amanullah et al., 2020).

Until now, some genetic basis studies identified the key genes regulating pedicel length trait in a few crops but not exactly for the weight and diameter. The first cytokinin oxidase (*CKX*) gene “*ZmCKX1*” was identified in maize crop (Houba-H' erin et al., 1999). The enzymatic activity of *CKX* related gene was then reported to encode the likely effects of growth hormones which promoted the pedicel length in tobacco (Paces et al., 1972). However, *CKX* encoded proteins are differentially reported by their small-size gene families in different plant species (Werner et al., 2006). Later, this *CKX* gene family has been well-characterized due to the more re-sequenced genomes, e.g., *Arabidopsis* (7 genes), rice (11 genes), maize (13 genes), and wheat (13 genes) (Lu et al., 2015). In cucumber, *CKX* depicted the regulation of endogenous cytokinin level which similarly controlled the pedicel length due to its cell number variation (Li et al., 2011). A set of cytokinin oxidase/dehydrogenase (*CKX*) encoding candidate genes has been identified that control the short internodal length and plant-dwarfness in muskmelon, watermelon, and dwarf wheat line “Tibetan” (Knauber and Banowetz, 1992; Jones and Schreiber, 1997; Banowetz et al., 1999; Zhang et al., 2019, 2021). The reduced *CKX* enzymatic activity in transgenic barley plants evidently showed the reduced transcription factor of spikelets and cause dwarf plant height as compared to the wild-type plant (Zalewski et al., 2010).

In addition, a controlled cell proliferation phenomenon during cell expansion has been reported. The cytokinin phytohormone along with *CKX*3 and *CKX*5 plays an antagonistic phenomenon in the reproductive development of meristem, larger inflorescence, and floral meristem tissues leading to increased fruiting rates and more yield of big size seed (Werner et al., 2003; Takashi et al., 2007). The absence of *HvCKX1*/*HvCKX9* in barley (Zalewski et al., 2014), *TaCKX6* in wheat (Zhang et al., 2012), and *OsCKX2*/*Gn1a* in rice (Ashikari et al., 2005) similarly induced the primary factor of root development (Gao et al., 2014) but the overexpression of *CKX7* triggers the pre-mature termination of root growth linked with the irregular development of vascular bundles (Kollmer et al., 2014). The *CKX3* gene upholds an active cytokinin level during the rhizosphere development and root formation; however, the *ljckx3* mutants display a suggestive decrease in root development and nodule formation (Reid et al., 2016). Recently, *CKX* showed likely effects of regulating the genetic variation in internal cell size and pedicel length of muskmelon (Cui et al., 2022).

In *Arabidopsis, KNAT1* and *ERECTA* genes were reported to play a key role in pedicel bending at the nodes of the *Arabidopsis* plant, and downward flower orientation was caused by the loss of chlorenchyma tissue in the abaxial regions (Douglas et al., 2002). In addition, the *KNAT2* and *KNAT6* genes showed a consistent role in the downward pedicel direction in *Arabidopsis* flowering (Ragni et al., 2008). The *ERECTA* genes depicted association with watermelon plant-dwarfism, and signified the regulatory effects for short internodal distances (Zhang et al., 2019). In tomato, the *BREVIPEDICELLUS (BP)* gene was found to regulate the cell proliferation during pedicel elongation which affects the abaxial region curvature (Wang et al., 2015). Further, the MADS-box gene encoding “*SHORT VEGETATIVE PHASE (NtSVP)*” exhibited a key role in pedicel elongation and orientation of tobacco (Wang et al., 2015). Then, *ARGONAUTE7 (S1AGO7)* gene was reported for regulation of upward direction of pedicel growth in tomatoes (Lin et al., 2016). *LEAFY* (LFY) gene function was explained in the pendent growth of *Arabidopsis* pedicels, and mutual function of LFY and BP showed reduction of cortical cell length in the abaxial domain (Yamaguchi et al., 2012).

Further, genes encoding *LEA* proteins were mainly found to regulate the post embryogenesis of cotton seed (Dure et al., 1981) and a total of 51 putative *LEA* protein family members (nine subfamilies) exhibit vegetative and reproductive tissue expression (Hundertmark and Hincha, 2008). Some *LEAs* depicted regulation of plant developmental stages instead of their key role of cell protection from the damage caused by water deficit regimes (Olvera-Carrillo et al., 2011), e.g., *SAG21* (*AtLEA5* and *At4g02380*) encode mitochondrial *LEA* protein and its antisense lines exhibited reduced primary root lengths, while over-expressed lines showed longer root hairs (Salleh et al., 2012). The rice gene *HVA1*, encodes a group 3 type small *LEA* protein, shown to promote the primary and lateral root elongation through the homeostasis regulation of signaling of auxin and abscisic acid (Chen et al., 2015).

In cucumber, the interaction of the *LEA*-encoding gene *CsFnl7.1* with *GLPs* found to regulate the increased FNLs in cucumber fruit, and germin-like protein *CsGLP1* similarly controlled the pedicel cell expansion (Xu et al., 2020). The *GLPs* also play an essential role in maintaining the cell dimension in rice (Banerjee and Maiti, 2010), cell wall expansion in cotton (Kim et al., 2004), cell growth in *Pinus caribaea* (Mathieu et al., 2003), and mediate cell expansion with auxin-dependent manner in *Prunus salicina* (El-Sharkawy et al., 2010). The *Arabidopsis* mutant phenotypes provided important insights into the functions of *DRPs* and *adl1A* (same as *drp1A*) and *adl1E* (same as *drp1E*) showed double mutations that resulted in embryo lethality with disturbed cytokinesis and cell expansion (Kang et al., 2003). It was also observed that the widespread defects in endocytosis, cellulose synthesizing, cytokinesis, and cell expansion in the Arabidopsis *rsw9* (radial swelling) mutant were caused by mutation in *DRP1A* (*At5g42080*) (Collings et al., 2008).

In the present study, we predicted a total of 454 genes in 5 delimited regions of flanking QTL regions, as indicated by generic gradients in circle chart (Figure 8). All the identified genes exhibited mostly non-synonymous SNPs in the CDS coding regions by showing the different proportions of changing the amino acid sequences in comparative parental lines (Supplementary Tables S4–S8). Our defined genes and their functional GO annotation exhibited the relevance with few earlier reported candidate genes encoding various protein families related with pedicel traits, e.g., the results of late embryogenesis abundant (*LEA*), cytokinin riboside, cytokinin dehydrogenase, IAA-amino acid, auxin-responsive, GDSL esterase/lipase, ABSCISIC ACID-INSENSITIVE 5-like protein, and germin-like protein family exhibited the key functions of suggestive genetic regulation of cell proliferation, cell elongation, and mitotic functions in many crops, but especially in Arabidopsis, cucumber, and muskmelon (Dure et al., 1981; Knauber and Banowetz, 1992; Jones and Schreiber, 1997; Banowetz et al., 1999; Kang et al., 2003; Werner et al., 2003; Takashi et al., 2007; Hundertmark and Hincha, 2008; Li et al., 2011; Olvera-Carrillo et al., 2011; Salleh et al., 2012; Lu et al., 2015; Reid et al., 2016; Song et al., 2016; Xu et al., 2020; Cui et al., 2022). Further, our cytological observations among both parent lines and their mapping populations also represented the differences in cell numbers and visual cell size, potentially highlighting that there must be major genetic regulatory mechanism between these defined genes and FP traits. So, we hypothesized that our defined genes would exhibit their key regulatory functions; however, further molecular work is required to validate these preliminary genetic findings and in-depth gene functional analysis.

## Conclusion

The present molecular experiment demonstrated the feasibility of next-generation sequencing- based whole-genome re-sequencing for the development of SNP markers, linkage mapping, and genetic loci mapping, by using the F_2:3_ derived mapping population. Herein, a total of 6 QTLs (5 major-effects and 1 minor-effect) were mapped between the delimited flanking regions in kb intervals and predicted some potential annotated genes. We first time reported the QTLs/genes regulating the FP-related traits (FPL, FPW, and FPD) of watermelon and no prior study has been reported yet. In crux, our preliminary QTL mapping results suggested that there might be a different range of mechanisms accountable for genetic control of FP traits. However, further fine genetic mapping and candidate gene expression profiling will lead to the understanding of multiple transcription factors and prominent genetic regulations of these traits.

## Data Availability Statement

The original contributions presented in the study are included in the article/Supplementary Material, further inquiries can be directed to the corresponding author/s.

## Author Contributions

SA planned and performed the molecular genetic experiment, data curation, formal analysis, prepared manuscript draft, and reviewed and edited the manuscript. BO and TY helped in field experiment. SheL and FA helped in formal analysis. ShiL and XW provided field research resources. ShuL and ZS provided the seed resources. PG and FL supervised the research project and reviewed and edited the manuscript. All authors approved the final manuscript version.

## Funding

This research was financially supported by Taishan Industrial Leading Talents Project (No. LJNY202112) and China Agriculture Research System of MOF and MARA (No. CARS-25).

## Conflict of Interest

ShuL and ZS were employed by company Shouguang Sanmu Seed & Seedling Co., Ltd. The remaining authors declare that the research was conducted in the absence of any commercial or financial relationships that could be construed as a potential conflict of interest.

## Publisher's Note

All claims expressed in this article are solely those of the authors and do not necessarily represent those of their affiliated organizations, or those of the publisher, the editors and the reviewers. Any product that may be evaluated in this article, or claim that may be made by its manufacturer, is not guaranteed or endorsed by the publisher.

## Supplementary Material

Supplementary Figure 1 Primary phenotypes of watermelon fruit pedicel (FP) traits of F_2:3_ mapping population at 5 mm scale.

Supplementary Figure 2 Scatterplot of distributed SNP-CAPS markers and collinearity test across the developed genetic linkage map of watermelon. The x-axis denotes the genetic position (cM) and y-axis denotes the physical position (bp) of whole genome markers.

Supplementary Table S1 Total mapped coverage of re-sequenced reads of two watermelon parent lines onto the reference genome (97103, v2).

Supplementary Table S2 Summary of detected SNP variants distribution in re-sequenced parental lines of watermelon.

Supplementary Table S3 Analysis of descriptive statistics of FP trait in parent lines, F_1_, and F_2_ derived F_3_ mapping population of watermelon.

Supplementary Table S4 List of predicted genes (116) in QTL region of *qFPD2.1* positioned on Cla97Chr02.

Supplementary Table S5 List of predicted genes (125) in QTL region of *qFPD2.2* positioned on Cla97Chr02.

Supplementary Table S6 List of predicted genes (98) in QTL region of *qFPD8.1* positioned on Cla97Chr08.

Supplementary Table S7 List of predicted genes (47) in QTL region of *qFPD10.1* positioned on Cla97Chr10.

Supplementary Table S8 List of predicted genes (68) in co-localized QTL regions of *qFPL4.1* & *qFPW4.1* positioned on Cla97Chr04.
