# Supplementary material for: Development of Whole Genome SNP-CAPS Markers and Preliminary QTL Mapping of Fruit Pedicel Traits in Watermelon
Source: Front Plant Sci. 2022 May 9;13:879919. doi: 10.3389/fpls.2022.879919 (PMC9128861; doi:10.3389/fpls.2022.879919)
Supplement: Supplementary Figure 1 — Primary phenotypes of watermelon fruit pedicel (FP) traits of F2:3 mapping population at 5 mm scale. [file Data_Sheet_1.ZIP › Supplementary Material/Supplementary Figure.docx]

**SUPPLEMENTARY FIGURES**


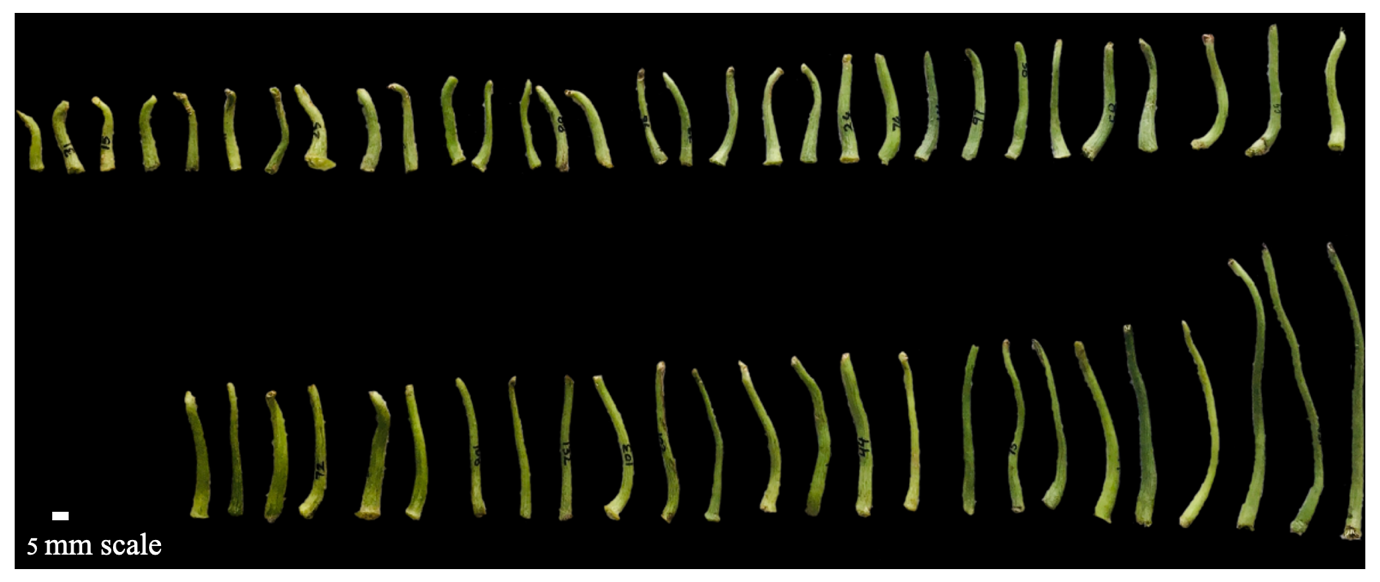


**SUPPLEMENTARY FIGURE 1 |** Primary phenotypes of watermelon fruit pedicel traits of F_2:3_ mapping population, at 5 mm scale.


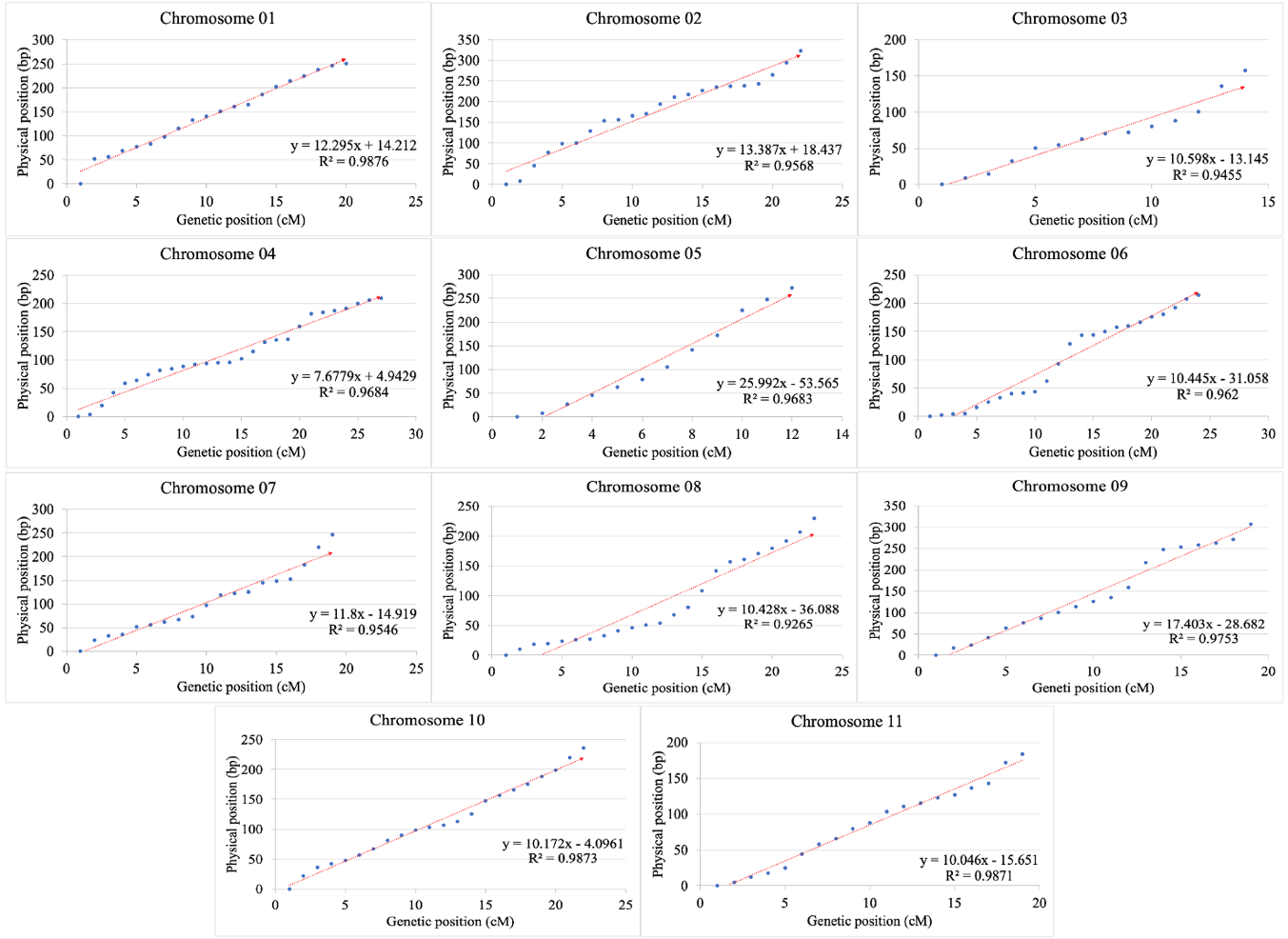


**SUPPLEMENTARY FIGURE 2** | Scatterplot of distributed SNP-CAPS markers and collinearity test across the developed genetic linkage map of watermelon. The x-axis denotes the genetic position (cM) and y-axis denotes the physical position (bp) of whole genome markers.
